# The Effectiveness of Double Cervical Cerclage and Bed Rest in Managing Complete Cervical Incompetence During the Second Trimester: A Case Presentation

**DOI:** 10.7759/cureus.101372

**Published:** 2026-01-12

**Authors:** Dimitrios Christakopoulos, Andreas Dimopoulos, Alexandros Trompoukis, Eleni Tsiampa

**Affiliations:** 1 2nd Department of Obstetrics and Gynecology, Elena Venizelou General and Maternity Hospital, Athens, GRC; 2 2nd Department of Obstetrics and Gynaecology/In Vitro Fertilization, Elena Venizelou Maternity Hospital, Athens, GRC; 3 Obstetrics and Gynaecology, General Hospital of Helena Venizelou, Athens, GRC

**Keywords:** bed rest, cervical insufficiency, double cervical cerclage, emergency cerclage, high-risk pregnancy, in vitro fertilization, obstetric case report, preterm birth prevention, shirodkar cerclage

## Abstract

Cervical insufficiency is a known cause of recurrent pregnancy loss and preterm delivery. Although cervical cerclage is the primary management option, some patients require additional reinforcement due to cervical failure.

This report describes a 45-year-old woman with a history of 5 second-trimester miscarriages who conceived through in vitro fertilization (IVF) using a donor oocyte. A prophylactic Shirodkar cerclage was placed at 12 weeks of gestation. Despite this, an ultrasound at 23 weeks showed funneling and almost complete loss of cervical length. A second emergency Shirodkar cerclage was inserted. The patient was hospitalized, maintained in the Trendelenburg position, and treated with tocolytics, antibiotics, progesterone, corticosteroids, and thromboprophylaxis while on strict bed rest. Cervical stability and fetal well-being were closely monitored until term. At 37 weeks and four days, a cesarean section was performed due to breech presentation after removal of both sutures, resulting in the delivery of a healthy female infant.

This case demonstrates that a second cervical cerclage can successfully maintain pregnancy up to term in cases of complete cervical insufficiency when the initial suture fails. The favorable outcome suggests that, in carefully selected patients, a double cerclage combined with comprehensive supportive management may help achieve term delivery. Although bed rest was implemented, its specific contribution remains uncertain and should be viewed as an adjunctive rather than a definitive therapeutic measure.

## Introduction

Cervical insufficiency, characterized by painless cervical dilation and recurrent pregnancy loss in the mid-trimester, complicates approximately 1% of pregnancies [1]. It is particularly common among women with previous second-trimester losses or cervical trauma. The condition is traditionally managed with cervical cerclage, most often using the McDonald or Shirodkar technique, to reinforce the cervix mechanically and prevent premature dilation [2].

In some patients, however, a single cerclage may not maintain cervical competence. Failure may occur due to tissue weakness, infection, or funneling above the suture line. Under such circumstances, a second or “repeat” cerclage can be considered. Evidence supporting this approach is limited to isolated case reports and small case series, but these experiences suggest that, in carefully selected women, a repeat procedure may avert pregnancy loss [3-4]. Repeat cerclage carries potential risks, including infection, cervical laceration, membrane rupture, bleeding, and preterm labor, and there are no standardized criteria to guide patient selection or procedural timing. As a result, this strategy remains controversial, particularly in women with multiple compounding risk factors. Alternative strategies after failure of a transvaginal cerclage include transabdominal cerclage, which has been associated with favorable outcomes in selected patients [5-6]. The present report describes a woman of advanced maternal age who conceived through assisted reproduction and underwent two Shirodkar cerclages during the same pregnancy, ultimately delivering a healthy term infant.

## Case presentation

A 45-year-old nulliparous woman was referred at 12 weeks of gestation with a history of 5 unexplained second-trimester miscarriages (other known causes of recurrent pregnancy loss had been excluded). The current pregnancy was conceived through IVF using a donor oocyte and the transfer of a single embryo.

Baseline transvaginal ultrasonography demonstrated a cervical length of 30 mm without funneling. Given her extremely high-risk obstetric history, a prophylactic Shirodkar cerclage was placed at 12 weeks of gestation under regional anesthesia. Postoperatively, the patient was advised to have partial bed rest and was started on vaginal progesterone (200 mg daily) and oral magnesium sulfate to reduce uterine activity.

**Figure 1:**
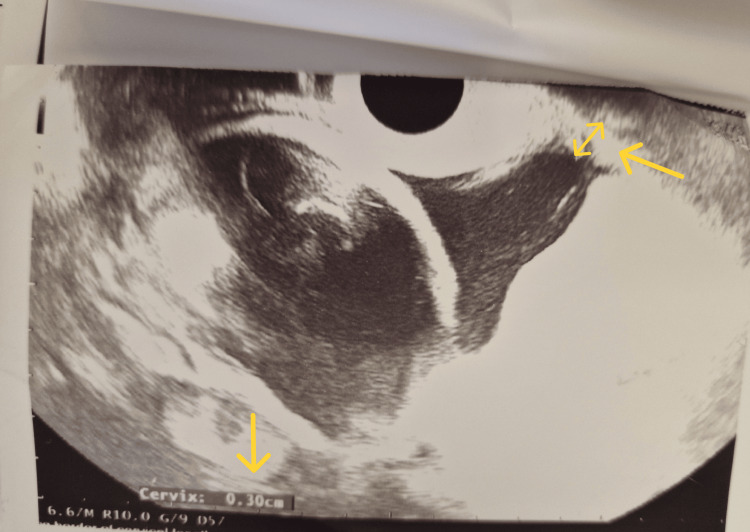
Transvaginal ultrasound at 23 weeks revealing funneling and shortening of cervical length (arrows)

At 23 weeks, follow-up ultrasonography showed U-shaped funneling and a functional cervical length of 3 mm, although the external os remained closed. No interval ultrasonography was performed between the initial 12-week scan and the 23-week follow-up, as the patient remained asymptomatic (Figure 1).

After detailed counseling and multidisciplinary discussion, management options were reviewed, including expectant management, transabdominal cerclage, and repeat transvaginal cerclage. Transabdominal cerclage was considered less appropriate due to the advanced gestational age, increased surgical invasiveness, and the absence of cervical dilation or membrane prolapse. A repeat emergency transvaginal Shirodkar cerclage was therefore selected as the most feasible and least invasive option.

Postoperatively, the patient developed uterine contractions. Laboratory testing revealed a mildly elevated C-reactive protein (CRP) level of 10 mg/L (reference <5 mg/L), while vaginal cultures were negative. She was treated with intravenous atosiban (for 48 hours) for tocolysis. Prophylactic broad-spectrum antibiotic (Cefoxitin) was administered following the second cerclage and discontinued after one week, once C-reactive protein (CRP) levels normalized, and vaginal cultures remained negative. Given her reduced mobility, low-molecular-weight heparin was administered for thromboprophylaxis, and the patient was maintained in a Trendelenburg position during the immediate postoperative period.

The patient continued intramuscular and oral progesterone, magnesium sulfate, iron, folic acid, and calcium supplementation. Dietary counseling was provided to prevent constipation and avoid straining. A course of antenatal corticosteroids (betamethasone, two doses of 12mg, 24 hours apart) was administered at 25 weeks of gestation for fetal lung maturity, with a repeat course at 28 weeks.

Serial ultrasonographic examinations demonstrated stable cervical findings and appropriate fetal growth. Doppler ultrasound at 33 weeks and repeat vaginal cultures at 35 weeks were unremarkable. At 37 + 4 weeks of gestation, following removal of both cerclage sutures, an elective cesarean section was performed due to persistent breech presentation. A female neonate weighing 2,490 g was delivered with Apgar scores of 10 at 1 and 5 minutes. The newborn required no neonatal intensive care. Both mother and infant were discharged in good condition. The timeline of clinical events is summarized in Table 1.

**Table 1:** Timeline of clinical events

Gestational Age	Clinical Findings	Intervention / Management
12 weeks	Cervical length 30 mm, no funneling	Prophylactic Shirodkar cerclage under regional anesthesia; initiation of vaginal progesterone and oral magnesium sulfate
23 weeks	U-shaped cervical funneling; functional cervical length 3 mm; closed external os	Emergency repeat transvaginal Shirodkar cerclage after counseling
Post–second cerclage	Uterine contractions; CRP 10 mg/L; negative vaginal cultures	Atosiban for tocolysis; prophylactic antibiotics; low-molecular-weight heparin; Trendelenburg positioning
25 weeks	Risk of preterm birth	First course of antenatal corticosteroids (betamethasone)
28 weeks	Ongoing high-risk pregnancy	Repeat course of antenatal corticosteroids
33 weeks	Normal fetal growth	Normal Doppler ultrasound
35 weeks	No signs of infection	Normal vaginal culture
37 + 4 weeks	Breech presentation	Removal of cerclage sutures; elective cesarean section
Delivery	Female neonate, 2,490 g	Apgar scores 10 at 1 and 5 minutes; no NICU admission

## Discussion

This case highlights the potential value of sequential cervical cerclage in maintaining pregnancy when an initial stitch fails. Although the technique is rarely required, it can be life-saving for pregnancies otherwise at imminent risk of loss. Success relies on timely diagnosis, absence of infection, and meticulous postoperative care. Previous literature offers limited but encouraging evidence. Sharma et al. described a case in which two Shirodkar cerclages were performed during one pregnancy, resulting in a term birth [4]. Damalie et al. presented a patient who underwent double cerclage placed simultaneously rather than sequentially, with successful prolongation of pregnancy to term [7]. Unlike these reports, our case involved a sequential approach, where a second suture was placed only after the initial one failed sonographically. Ru et al. reviewed a small series of repeat cerclages and observed that pregnancy duration was extended by an average of eight weeks, although some women still delivered preterm [3]. The gestational prolongation achieved in our case--extending to 37 weeks--surpasses the durations reported in that study. Additional evidence supporting surgical rescue comes from reports describing the use of transabdominal techniques [5-6]. Collectively, these cases emphasize that when the cervix remains structurally compromised, further mechanical reinforcement, whether transvaginal or transabdominal, may safely sustain gestation. Our patient differed from most published cases due to her advanced maternal age, IVF conception, and nearly complete funneling at 23 weeks, factors typically associated with poor prognosis. The combination of surgical precision, infection prevention, and pharmacologic support was crucial to maintaining uterine quiescence and allowing fetal maturity. An additional feature of this case was the implementation of strict bed rest following the second procedure. The role of bed rest in preventing preterm birth has long been debated. Indeed, larger analyses, including a Cochrane review, have not demonstrated consistent benefit and have drawn attention to the potential harms of immobilization, such as thromboembolism, muscular atrophy, and emotional distress [8]. Finally, McCall et al. questioned the ethical basis of prescribing prolonged bed rest in the absence of evidence [9]. In the present case, bed rest was introduced as a supportive adjunct rather than a primary therapeutic measure. The patient’s immobility was managed proactively with anticoagulation to minimize risk, and close inpatient monitoring ensured early detection of complications. It is therefore likely that the term outcome resulted from a multifactorial approach, including surgical reinforcement, infection prevention, uterine relaxation, and vigilant follow-up, rather than from bed rest alone.

In conclusion, it is important to acknowledge that, in this case, multiple interventions, including sequential Shirodkar cerclages, progesterone supplementation, tocolysis, and bed rest, were implemented. The relative contribution of each to the favorable outcome cannot be determined. Furthermore, this report describes a single patient; therefore, causality cannot be inferred, and the findings should be interpreted cautiously. Nevertheless, this case highlights the potential role of repeat transvaginal cerclage in carefully selected high-risk patients, while underscoring the need for further studies to evaluate efficacy and safety.

## Conclusions

Sequential cervical cerclage may represent a valuable rescue option for women with cervical insufficiency when the initial stitch fails, even in the context of advanced maternal age or assisted reproduction. The present case demonstrates that a second Shirodkar cerclage, combined with strict bed rest, infection prevention, uterine quiescence, thromboprophylaxis, and comprehensive monitoring, may support continuation of pregnancy to term in selected cases. While strict bed rest was part of the management plan, current scientific evidence does not confirm a direct causal role in improving pregnancy outcomes. Its use should therefore be individualized, reserved for select situations, and accompanied by preventive strategies against immobilization-related complications.

## References

[1] Brown R, Gagnon R, Delisle MF (2013). Cervical insufficiency and cervical cerclage. J Obstet Gynaecol Can.

[2] Simcox R, Shennan A (2007). Cervical cerclage: a review. Int J Surg.

[3] Ru P, Ni X, Xu W (2024). Perinatal outcomes in patients undergoing repeat cerclage: a retrospective case series study. Int J Gynaecol Obstet.

[4] Sharma N, Ram K, Sharma A (2013). Fetal outcome in repeat cervical encirclage in same pregnancy. Int J Reprod Contracept Obstet Gynecol.

[5] Moria A, Aljaji N, Miner L, Tulandi T (2011). Abdominal cerclage after failed transvaginal cervical cerclage. Gynecol Surg.

[6] Hall DR, van de Vyver M (2023). Transabdominal cerclage during pregnancy: a retrospective single operator series over a quarter century. Int J Gynaecol Obstet.

[7] Damalie FJ, Senaya CM, Dassah ET, Konney TO, Ameyaw E, Larsen-Reindorf R, Asubonteng GO (2024). Double cervical cerclage for cervical insufficiency: case report. Afr J Reprod Health.

[8] Sosa CG, Althabe F, Belizán JM, Bergel E (2015). Bed rest in singleton pregnancies for preventing preterm birth. Cochrane Database Syst Rev.

[9] McCall CA, Grimes DA, Lyerly AD (2013). "Therapeutic" bed rest in pregnancy. Unethical and unsupported by data. Obstet Gynecol.

